# Remote W.A.R.A. Compared With Face-to-Face W.A.R.A.: A Pilot Study

**DOI:** 10.3389/fpsyg.2020.620027

**Published:** 2021-01-15

**Authors:** Paula Weerkamp-Bartholomeus, Donatella Marazziti, Therese van Amelsvoort

**Affiliations:** ^1^Department of Psychiatry and Neuropsychology, School for Mental Health and Neuroscience, Maastricht University, Maastricht, Netherlands; ^2^ReAttach Therapy International Foundation, Voerendaal, Netherlands; ^3^Dipartimento di Medicina Clinica e Sperimentale, Section of Psychiatry, University of Pisa, Pisa, Italy

**Keywords:** remote therapy, ReAttach, affect regulation, emotion regulation, Internet-based therapy, W.A.R.A.

## Abstract

**Background:**

Since the outbreak of the COVID-19 pandemic and its social restriction measures, online therapy is a life-saving possibility for patients with acute stress. Wiring Affect with ReAttach (W.A.R.A.) is a brief psychological intervention aiming to decrease negative affect, that can be offered online.

**Methods:**

We assessed the effect of remote W.A.R.A. on negative affect in 37 patients. Consequently, we compared the effect of remote W.A.R.A. versus face-to-face W.A.R.A on negative affect in a cross-sectional design.

**Results:**

W.A.R.A. remote therapy provoked a significant reduction of negative affect with a large effect size (*d* = 3.08, *p* < 0.001). However, the reduction on negative affect was smaller than with W.A.R.A. face-to-face. We found a substantial difference between W.A.R.A. remote therapy and W.A.R.A. face-to-face in decrease of negative affect (*d* = 1.36, *p* < 0.001).

**Limitations:**

The major limitation of the pilot-study is the sample size of 37 patients. Besides, we designed a numeric rating scale for evaluating negative affect. We investigated the impact on negative affect by assessing “unpleasant feelings.” This conceptualization of negative affect might still be a point of discussion.

**Conclusion:**

The study’s findings indicated that W.A.R.A. remote therapy significantly reduced negative affect, but to a lesser extent than W.A.R.A. face-to-face. Nevertheless, W.A.R.A. remote therapy might offer a fast relief, especially when personal contact is difficult.

## Introduction

The COVID-19 outbreak is an urgent concern for mental health around the world, in addition to the threat to physical health. The pandemic’s impact on psychological and physical health will be devastating unless we provide our patients with a therapy or self-regulation strategy to dampen the acute stress response (Porges, 2020). Indeed, in times of crisis, it is of utmost importance to provide psychological support as soon as possible (Marazziti, 2020; Srivastava, 2020).

Accessible forms of psychotherapy that can help to alleviate the initial psychological distress may prevent the development of post-traumatic stress disorders, obsessive-compulsive disorders, depression, and social anxiety (Di Giuseppe, 2020; Mucci, 2020; Porges, 2020). Online therapy is a life-saving possibility to help patients deal with psychological distress without violating the social restrictions imposed in almost all countries to limit the diffusion of the virus.

By providing online self-regulation strategies, such as Wiring Affect with ReAttach (W.A.R.A.) remote therapy, psychologists might be able to guide patients into the downregulation of their psychological distress. Hence, as soon as the COVID-19 pandemic began, we started to give free online W.A.R.A. remote therapy courses to provide this potential first-aid psychological intervention to professionals worldwide (Weerkamp-Bartholomeus, 2020b).

Wiring Affect with ReAttach is a brief psychological intervention, generally provided by trained ReAttach therapists for patients struggling with persistent complaints of negative feelings and sensory over-responsivity, aimed to decrease negative affect (Weerkamp-Bartholomeus, 2019, 2020a). According to recent studies, patients with AD(H)D, autism, post-traumatic stress, chronic pain, and traumatic brain-injury commonly experience sensory over-responsivity as a comorbid condition (Greenspan et al., 1998; Bundy et al., 2008; Lillas et al., 2018; Pfeiffer et al., 2018; Roberts et al., 2018; Schaaf et al., 2018; Delahooke, 2019; Porges et al., 2019; Christensen, 2020).

Wiring Affect with ReAttach refers to “Wiring Affect with ReAttach” because the exercise is part of the extended ReAttach procedure, which we will briefly describe. ReAttach is an accessible, tailored, transdiagnostic intervention based on the activation of healthy development, aiming to help children and adults to become the best possible version of themselves (Bartholomeus, 2013) and contains elements from evidence-based interventions, such as Ayres Sensory Integration (ASI) training (Schaaf et al., 2018; Schoen et al., 2019; Abelenda and Rodríguez Armendariz, 2020), Play (Chang et al., 2019; Giacomucci and Marquit, 2020), Social Cognitive Training (Haut et al., 2019; Miley et al., 2019), Cognitive Bias Modification (Klein et al., 2018; Kemps et al., 2019; Zhang et al., 2019), Mindfulness-Based Cognitive Therapy (Tickell et al., 2020), and Compassion-Focused Therapy (Sommers-Spijkerman et al., 2018). ReAttach aims to change information-processing biases such as negativism and training new adaptive cognitive processes to (re)gain coherence in terms of realistic concepts of the self, (significant) others, and the world. ReAttach achieves these changes by modifying arousal and sensory stimuli, social cognitive training, and associative learning. ReAttach is an emotional-neutral experience and gentle non-invasive therapy. ReAttach aims to treat mental health problems by targeting proven transdiagnostic processes while using a standard protocol. The uniqueness of ReAttach lies in the targeting of multiple underlying core processes, simultaneously and in a fixed order in one fluent therapy session: optimization of physical arousal, sensory processing, conceptualization, mentalization, associative learning, and associative memory formation.

Wiring Affect with ReAttach is made up of essential elements of ReAttach, such as arousal regulation, sensory stimulation, multiple sensory processing, and associative memory formation (Bartholomeus, 2013; Weerkamp-Bartholomeus, 2020b). During this W.A.R.A. exercise, ReAttach therapists specifically aim at wiring negative affect by simultaneous activation of multiple associations under ReAttach conditions (Weerkamp-Bartholomeus, 2019). Instead of focusing on emotional or physical pain, in W.A.R.A., the therapists work with general unpleasant feelings for which there are no words yet. When W.A.R.A. is provided face-to-face, the therapist externally regulates the patient’s arousal and sensory processing and requires the therapist’s proximity and physical contact through the gentle tapping on the patient’s hands. In W.A.R.A. remote therapy, the therapist instructs the patient to self-regulate the arousal and sensory processing by verbal instructions and exercises. In both cases, the goal of W.A.R.A. is to conceptualize and store unpleasant feelings through sensory integration using associative memory formation.

Due to its accessibility and simplicity, W.A.R.A. might serve as a self-regulation tool provided by remote therapy.

Although previous studies assessed the effectiveness of W.A.R.A. performed by a therapist through face-to-face contact, no research has yet been conducted into the application of W.A.R.A. delivered online. We examined the efficacy of remotely delivered W.A.R.A. in the reduction of negative affect and compared the results of the W.A.R.A. remotely provided with results of W.A.R.A. face-to-face by a therapist.

## Subjects and Methods

### Study Design

In the period of lockdown, Dutch qualified ReAttach therapists, professionally educated in psychology, occupational or physical therapy, offered 37 patients with stress-related complaints (men 27%, women 73%; mean age + SD 47.6 + 18.7 years) online guidance. These ReAttach therapists all participated in the free online W.A.R.A remote therapy training. Since ReAttach therapy is not suitable for online treatment, they offered W.A.R.A. remote therapy as a guided self-regulation tool to decrease negative affect as part of online consultation. Reported suicidality risk and alcohol or drug abuse at the time of the online consultation were exclusion criteria for W.A.R.A. remote therapy according to the standard ReAttach procedures (Bartholomeus, 2013; Weerkamp-Bartholomeus, 2020a).

The data were collected as part of routine clinical care, and therefore no research ethical committee approval was necessary. All the patients consented to data use for research purposes. Online assessment took place before and after W.A.R.A. remote therapy referring to one online video consultation.

Information about patients’ diagnoses and medication is listed in Table 1. A within-subjects design was used to assess the efficacy of W.A.R.A. remote therapy in this group of 37 patients. To compare the efficacy of W.A.R.A. remote therapy versus W.A.R.A. face-to-face we used a cohort of 46 patients (men 30%, women 70%; mean age + SD 43.3 + 13.3 years) from a previous study who received W.A.R.A. face-to-face (Weerkamp-Bartholomeus et al., 2020). We assessed the comparability of both groups on the distribution of age, gender and base-line negative affect score and found no significant differences between groups. Both groups of patients experienced problems in the regulation of stress. All data were sampled as part of care as usual, and therefore permission from the medical ethics review committee was not required. The study was carried out by ReAttach Therapy International Foundation in the Netherlands.

**TABLE 1:** Sample description of patients (*N* = 83).

	Face-to-face	Remote
**Demographic patient’s characteristics**		
Sample size, *N*	46	37
Gender, male in %	30	27
Age, *M* (SD)	43.3 (13.3)	47.6 (18.7)
Age, min–max	17–68	13–87
**DSM-5 diagnoses, frequency (N)**		
AD(H)D	2	1
Anxiety disorder	1	
Autism spectrum disorder		1
Depression	3	1
Eating disorder	1	
Personality disorder	1	
PTSD	3	1
Somatic symptom disorder		1
**Other syndromes, frequency (N)**		
Burnout	4	
Chronic fatigue	3	
Chronic pain		1
Diabetes		1
Functional neurological disorder	1	
Sensory processing disorder	5	
Tinnitus	1	
Traumatic brain injury		1
**Medication, frequency (N)**		
Antidepressant	1	
Stimulants	2	
Sedatives		1
Analgesics	1	2

### Interventions

#### Wiring Affect With ReAttach

Qualified ReAttach therapists provided W.AR.A. conform trained protocol (Weerkamp-Bartholomeus, 2019, 2020a). Usually, a therapist regulates the participant’s arousal, sensory stimuli, and negative affect during W.A.R.A. This requires physical proximity of the therapist: W.A.R.A. face-to-face. In this study, we compared the results of a group of 46 patients who received W.A.R.A. face-to-face with a group of 37 patients who received W.A.R.A. remote therapy.

For the remote therapy we simplified the W.A.R.A. instruction. By remote instruction, the patient needs to adopt several skills that the therapist normally performs, such as sensory stimulations (tapping) and arousal regulation by change of tapping speed. Therefore, we chose to simplify the tapping technique which we normally use for the downregulation, to gently pressing on a surface. At first the patients learned how to optimize arousal for multiple sensory processing and dampen unpleasant feelings through tactile stimulation. The fast tactile stimulation at the beginning of W.A.R.A. and during the instruction of memory formation, remained the same. The students of the W.A.R.A. remote therapy course learned to tap fast as soon as they heard the word “tap” and downregulate as soon as they heard “stop.” During W.A.R.A. face-to-face, the therapist delivers a tailored intervention by adapting the voice and choice of positive concepts that appeal to the patient. During the W.A.R.A. remote therapy course, no such adjustment was made. We decided to offer every professional the same video instruction with similar positive concepts during low-frequency tapping (music, dance, favorite meal, enthusiasm, love, gratitude, and the sun). Also, we offered recorded voice instructions on-line as an aid for W.A.R.A. self-regulation.

### Procedure

All patients received the same questionnaire to score negative affect before and after the intervention. We conceptualized negative affect as “unpleasant feeling,” rated on an 11-point numeric rating scale developed for by us. At baseline, the patients rated an unpleasant feeling on a scale of 0 (not unpleasant at all) to 10 (most unpleasant). After this baseline measurement, they received the intervention with was either W.A.R.A. face-to-face or W.A.R.A. remote therapy. After the intervention, we immediately asked the patients if they could still feel this unpleasant feeling: yes or no. Finally, we asked these patients to re-engage with the negative affect and again rate the unpleasantness.

### Data Analysis

We used descriptive statistics and a Consort Flow-chart to contextualize the demographic characteristics and the flow of the patients. With the Wilcoxon Signed Rank Test, we compared the baseline and post-test negative affect ratings within-subjects. We used one-way ANOVA for comparison of demographic characteristics and mean affect rating at baseline between groups. To assess the differences in outcome between groups, we used the Mann–Whitney U test. Pairwise comparisons were performed (IBM Corp, 2012). The statistical analyses were two-sided, and we set the significance level at 5%. We reported the interpretation of effect sizes conform Cohen (1992). To analyze the data, we used the Statistical Package for Social Science (S.P.S.S.) version 22 (Armonk, NY, United States) (IBM Corp, 2012).

## Results

### Descriptive Statistics

**FIGURE 1:**
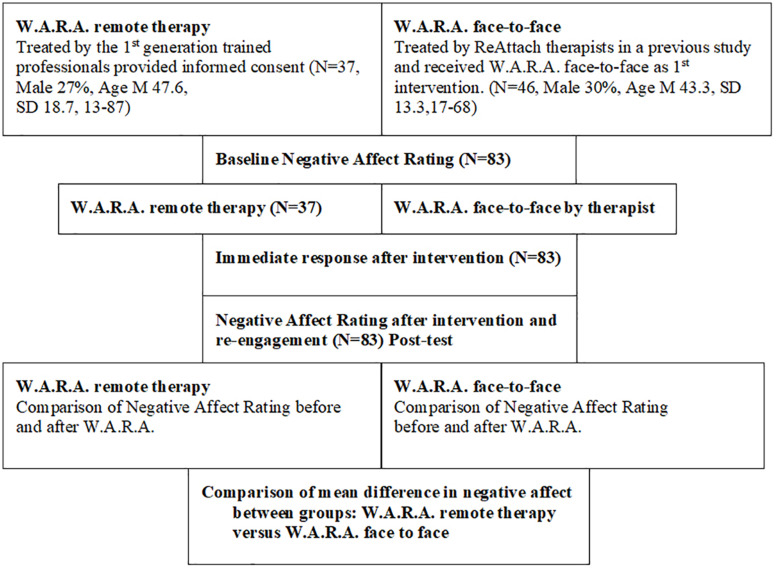
CONSORT flow diagram and demographics of the cross-sectional cohort study.

Figure 1 and Table 1 present the patient flow and descriptive characteristics of the cross-sectional study. With one-way ANOVA, we assessed differences between the groups in age, gender, or baseline scores. There were no significant differences in age *F*(1,81) = 1.492, *p* = 0.225, gender *F*(1,81) = 0.113, *p* = 0.737 or baseline sore *F*(1,81) = 0.049, *p* = 0.825 between the W.A.R.A remote therapy group and the W.A.R.A. face-to-face group.

### Effect on Negative Affect Scores of W.A.R.A. Remote Therapy (*N* = 37)

**FIGURE 2:**
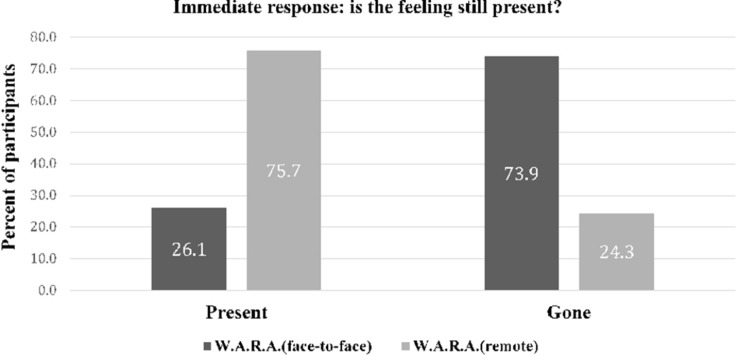
Immediate post intervention response of the patients after W.A.R.A. by remote therapy (*N* = 37), versus W.A.R.A. by therapist (*N* = 46).

**FIGURE 3:**
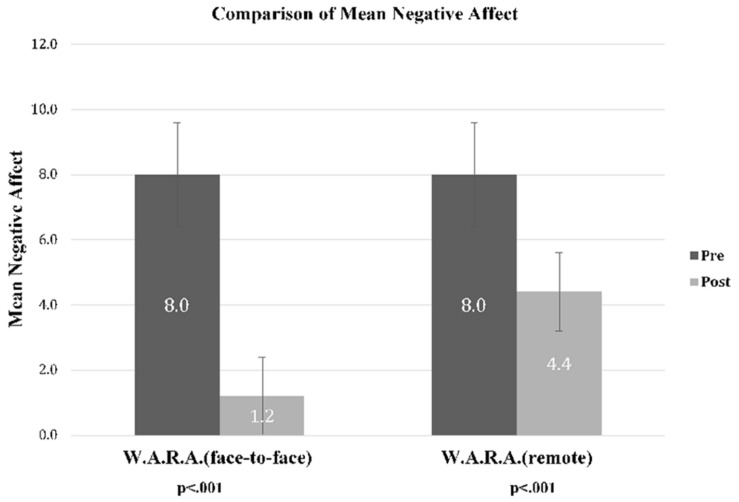
Mean negative affect scores, before and after W.A.R.A. face-to-face (*N* = 46) and W.A.R.A. remote therapy (*N* = 37).

As shown in Figure 2, immediately after online therapy W.A.R.A, 24.3% of the respondents reported that the negative affect was gone. As shown in Figure 3, at baseline, the mean negative affect score of the W.A.R.A. remote therapy was 8.0 (SD = 1.2). The mean negative affect score decreased to 4.4 (SD = 2.0) after re-engagement.

We found symmetrically distributed difference scores, as assessed by a histogram with a superimposed normal curve. Of the 37 patients of the present study, 34 patients reported a reduction of negative affect, and 3 reported no change. The Wilcoxon signed-rank test determined that there was a significant decrease in negative affect (Mdn = −4.00) after W.A.R.A. remote therapy (Mdn = 3.00), compared to the negative affect before the intervention (Mdn = 7.00), *z* = −5.10, *p* < 0.001. The effect size was large, *d* = 3.08 (Cohen, 1992). Evaluation of the remote intervention outcomes in terms of success (positive change) or failure (no change or negative change) resulted in a probability of success for W.A.R.A. remote therapy of 92%.

### Comparison W.A.R.A. Remote Therapy Versus W.A.R.A. Face-to-Face

We compared the data from the W.A.R.A. remote therapy group versus the data from 46 patients from a previous W.A.R.A. face-to-face by therapist study (Weerkamp-Bartholomeus et al., 2020). As shown in Figure 2, immediately after the remote instruction, 24.3% of the patients compared to 73.9% of the patients who received W.A.R.A. face-to-face reported that the negative affect was gone. In the group of patients who received W.A.R.A. face-to-face, the mean negative affect scores decreased to 1.2 after re-engagement compared to 4.4 in W.A.R.A. remote therapy. We run a Mann–Whitney U test to compare the reduction of negative affect of W.A.R.A. remote therapy versus W.A.R.A. face-to-face. Reduction of negative affect was larger after W.A.R.A. face-to-face (mean rank = 54.13) than after W.A.R.A. remote therapy (mean rank = 26.92), U = 293.000, *z* = −5.144, *p* < 0.001. The difference in reduction of negative affect was large *d* = 1.36 (*p* < 0.001).

## Discussion

This pilot study aimed to find out whether W.A.R.A remote therapy might represent a valuable first-aid psychological intervention. According to us, this is particularly relevant at this time of COVID-19 pandemic. During this uncertain and challenging time, stress, and anxiety increase because we are all concerned about the magnitude and effects of this crisis (Albott et al., 2020). In times wherein psychological consultation is restricted, online psychotherapy might offer a solution. From a preventive point of view, it is vital to support professionals and patients with self-regulation strategies at hard times, such as the present caused by the COVID-19 pandemic (Marazziti, 2020; Porges, 2020).

We examined the effect of W.A.R.A. remote therapy on negative affect and compared the reduction of negative affect of W.A.R.A. remote therapy versus W.A.R.A. face-to-face. W.A.R.A. remote therapy resulted in a significant decrease in negative affect with a large effect size. However, we found a larger reduction of negative affect after W.A.R.A. face-to-face compared to W.A.R.A. remote therapy.

We pose several potential explanations for the fact that W.A.R.A. remote therapy was less successful than the W.A.R.A. face-to-face. First of all, it is much easier for a therapist to influence complex transdiagnostic processes such as co-regulation of arousal and affect face-to-face than by online therapy. Furthermore, the timing, a crucial element during W.A.R.A., is more difficult to achieve by online guidance. We also think that the real proximity enhances the patient’s trust and might induce more positive expectations about the intervention outcome; W.A.R.A. face-to-face might induce more placebo-effect. Moreover, we believe that it is not apparent for patients with stress-related complaints to have either neutral or positive expectations about their self-regulation abilities. Their elevated stress-levels will more likely induce negative expectations, which may lead to nocebo-effects, especially without the physical presence and re-assurance of a therapist.

The results of this pilot study suggest that even for a brief and accessible self-regulation exercise as W.A.R.A., face-to-face interaction with a therapist is more effective. W.A.R.A. face-to-face is also more favorable, considering the various ethical arguments against engagement in online psychotherapy such as privacy, confidentiality, and emergency issues (Stoll et al., 2020).

The availability, rapid transferability, lightness of the intervention, and efficiency of W.A.R.A in decreasing negative affect should be strongly highlighted. The fact that the intervention can be used as a self-regulation tool also makes the application of W.A.R.A. remote therapy even more interesting. If we can actually train patients to use W.A.R.A. as a self-regulation technique, this will no doubt contribute to enhance their autonomy and self-control.

Wiring Affect with ReAttach remote therapy can be compared with other short-term online self-help interventions, such as Compassion-Focused Therapy (Gilbert, 2010, 2014; Hudson et al., 2019) and other mindfulness-based interventions (Lilly et al., 2019) focusing on the downregulation of psychological distress (Porges, 2020) and promotion of calmness (Cheng et al., 2020; Sulaiman et al., 2020). An advantage of W.A.R.A. compared to other therapies seems that negative affect wired during W.A.R.A. becomes less intense or is gone, making it hard to reengage with the previous unpleasant feelings. Thus W.A.R.A. might be helpful to stop rumination. Eye Movement Desensitisation and Reprocessing (EMDR) is another rapid intervention for posttraumatic stress disorders (PTSD) that can be provided online and shows promising results (Spence et al., 2013). However, the systematic review of Lenferink et al. (2020) concludes that online EMDR is still premature and, therefore, for patients with PTSD, online CBT is currently still preferable.

Since W.A.R.A. can be trained as a self-regulation technique, W.A.R.A.’s more frequent practice might improve stress-resilience in vulnerable patients. For more extended online therapies such as Online Group Schema Therapy Based Day-Treatment (van Dijk et al., 2020), W.A.R.A. remote therapy could be a welcome addition to teach patients with negative affect self-regulation. Even though the findings of this pilot study cannot be generalized to clinical populations, the results suggest that W.A.R.A. remote therapy has the potential as a brief first-aid psychological intervention.

We would acknowledge a criticism of the technique: W.A.R.A. remote therapy is beneficial as a strategy to deal with unpleasant feelings, sensory over-responsivity and psychological distress, but it cannot solve more severe mental health problems (Aldao, 2010). ReAttach therapists usually use W.A.R.A. as an additional tool during a treatment process to help patients reduce negative feelings for which there are no words yet. This exercise helps to regain self-control and to process unpleasant feelings very quickly. Therefore, W.A.R.A. is ideal for getting to know ReAttach, making ReAttach even more accessible. It should be noted that W.A.R.A. does not replace ReAttach as a schema therapy for adults and children or as a multimodal intervention for autism (Weerkamp-Bartholomeus, 2015, 2018). ReAttach, although also brief, is more extensive, which is necessary to, among other things, automate affect regulation, sensory integration, coherent conceptualization, and mentalization. W.A.R.A. is far too limited for treating complex problems, such as in patients with trauma, personality disorders, behavioral issues, or pervasive developmental disorders.

### Strength and Limitations

Wiring Affect with ReAttach is an innovative non-invasive psychological intervention by which negative affect can be reduced effectively.

This pilot study suffers from several limitations that should be mentioned, such as the small sample size and the design of the numeric rating scale, that was based on a numeric rating scale for the evaluation of pain (Williamson and Hoggart, 2005). Although previous research from Slaby (2019) and Krueger (2016) support our choice, the conceptualization of negative affect in a combination of arousal and unpleasant feelings might still be a point of discussion. More specifically, the question arises whether or not we can evaluate the reduction of such a wide variety of subjective unpleasant feelings.

In the near future, we would like to investigate the application of W.A.R.A. for specific patient groups. Since W.A.R.A. can be trained as a self-regulation technique, more frequent practice of W.A.R.A might improve stress-resilience in vulnerable patients. We think that it would be very interesting to investigate such W.A.R.A. training in a randomized controlled design with follow-up measurements.

## Conclusion

Wiring Affect with ReAttach, usually provide face-to-face by a ReAttach therapist, can successfully be provided as remote therapy and significantly reduce negative affect. Although W.A.R.A. face-to-face is more favorable and more effective, these first results of W.A.R.A. remote therapy are encouraging. Besides, W.A.R.A. is accessible, and online training for professionals is free. Furthermore, W.A.R.A. remote therapy can be learned as a self-regulation technique. Extended research with a larger sample size and with specific patient groups is needed to assess the full potential of W.A.R.A. face-to-face and W.A.R.A. remote therapy.

## Data Availability Statement

The raw data supporting the conclusions of this article will be made available by the authors, without undue reservation.

## Ethics Statement

Since data were collected as part of routine clinical care, no research ethical committee approval was necessary. All patients consented to data use for research purposes.

## Author Contributions

All authors contributed to the conception and design of the study. PW-B collected and analyzed the data and wrote the manuscript. All authors contributed to the manuscript revision, read, and approved the submitted version.

## Conflict of Interest

The authors declare that the research was conducted in the absence of any commercial or financial relationships that could be construed as a potential conflict of interest. The reviewer GO declared a shared affiliation, with no collaboration, with one of the authors DM to the handling editor at the time of the review.
