# Supplementary material for: Remote W.A.R.A. Compared With Face-to-Face W.A.R.A.: A Pilot Study
Source: Front Psychol. 2021 Jan 15;11:620027. doi: 10.3389/fpsyg.2020.620027 (PMC7844207; doi:10.3389/fpsyg.2020.620027)
Supplement: Supplementary file 1 [file Data_Sheet_1.PDF]

W.A.R.A. remote therapy protocol

W.A.R.A. takes about 90 seconds, and you can apply it anywhere, anytime when you feel bad. For vulnerable people who cannot independently deal with their negative affect, learning to self-regulate is a small, but crucial step forward.

Instruction W.A.R.A. remote therapy

Step 1: Participate in the free online W.A.R.A. remote therapy training.
Email paula@reattachacademy.nl or via reattachacademy.com

Step 2: Teach your patient how to regulate optimal physical arousal
The instruction for activation of optimal processing window is "tap," and the instruction for downregulation is "stop."

Step 3: Choose the concepts that you will use for the W.A.R.A.
Communicate with your patient so you can create your ensemble of at least five positive concepts, which all should be triggering intense positive emotions.

Step 4: Practice the verbal instruction
Verify that your patient exactly understands what to do.

Step 5: Practice the tapping
Check if your patient knows what to do.

Step 6: Co-regulate your patient's arousal
Verify that you have optimal arousal and joint attention

Step 7: Provide the following instructions by online guidance:
Spoken instruction between " "
Replace the concepts by the words you have chosen.

- **"Tap"**
- **"Go to the negative feeling and nod your head when you clearly feel it"**
- (*patient nods the head*)
- **"Stop"** *(immediately after saying stop proceed with the positive concepts)*
- **"Concept1, Concept2, Concept3, Concept4, Concept5"**
- *Be silent (give processing time for association)*
- **"Tap"**
- **"Remember this exercise"**
- **"Stop"**
- *Be silent (give processing time for integration and memory formation)*
- **"Tap"**
- **"You may open your eyes".**

Step 8: Evaluate the outcome with your patient
